# Swedish Consensus Recommendation for Follow‐Up After ICU Stay

**DOI:** 10.1111/aas.70361

**Published:** 2026-10-25

**Authors:** Eva Åkerman, Anna Schandl, Mona Ringdal, Oili Dahl, Lotti Orwelius

**Affiliations:** ^1^ Department of Perioperative Medicine and Intensive Care Karolinska University Hospital Stockholm Sweden; ^2^ Department of Health Sciences Lunds University Lund Sweden; ^3^ Department of Perioperative and Intensive Care Södersjukhuset Stockholm Sweden; ^4^ Department of Clinical Science and Education, Södersjukhuset Karolinska Institutet Stockholm Sweden; ^5^ Institute of Health and Care Sciences at Sahlgrenska Academy University of Gothenburg Gothenburg Sweden; ^6^ Department of Anaesthesia and Intensive Care West Hospital Kungälv Sweden; ^7^ Department of Neurobiology, Care Sciences and Society Karolinska Institute Stockholm Sweden; ^8^ Department of Anaesthesia and Intensive Care Linköping University Hospital Linköping Sweden; ^9^ Department of Biomedical and Clinical Sciences Linköping University Linköping Sweden

**Keywords:** consensus recommendation, critical care, follow‐up, post intensive care syndrome, post‐ICU

## Abstract

**Background:**

A substantial proportion of intensive care unit (ICU) survivors experience long‐term physical, psychological and cognitive impairments after critical illness. However, methods for identifying patients in need of post‐ICU support vary widely. As a result, many patients are left without access to established rehabilitation pathways. This consensus recommendation provides a framework for the organisation and content of follow‐up for adult ICU survivors in Sweden, informed by available evidence, stakeholder perspectives and expert consensus.

**Methods:**

The National System for Knowledge‐Driven Healthcare Management (NPO) in Sweden commissioned the development of a practice recommendation for the follow‐up of critically ill adults. Multidisciplinary experts in ICU survivorship formed a task force group. A systematic literature search in four databases was conducted in 2024 to identify pre‐existing systematic reviews on the topic. The literature was reviewed, assessed for quality and recommendations were developed based on the literature evidence. The draft was discussed among multiprofessional groups involved in follow‐up, in workshops with critical care nurses (*n* = 30) and professionals responsible for follow‐up clinics (*n* = 74) and among patient representatives (*n* = 4). The final draft underwent external peer review by representatives from relevant professional organisations, the national registry for intensive care and governmental agencies in Sweden.

**Results:**

The consensus recommendation suggests that all adult patients with an ICU length of stay ≥ 48 h are recommended for follow‐up, with an initial contact within 1 week of ICU discharge to assess clinical status, identify early problems and ensure continuity of care. Patients and their families are recommended to be offered an outpatient follow‐up visit at 2–3 months after discharge, led by ICU clinicians and, when possible, delivered by a multidisciplinary team. Validated screening tools and objective tests are recommended to assess relevant outcomes, including post‐traumatic stress, anxiety, depression, cognitive impairment, physical function and Health‐Related Quality of Life (HRQoL) as appropriate. Patients with identified problems should be referred to appropriate healthcare providers or specialist services for further management.

**Conclusion:**

This consensus recommendation provides a framework for patient selection, timing and assessment to support a more structured and equitable management of post‐intensive care syndrome.

**Editorial Comment:**

Systematic follow up of ICU survivors after hospital discharge is needed in order to assess long‐term patient‐centred outcomes. This is a consensus set of recommendations and not a GRADE‐based clinical practice guideline for doing this, which can be considered by relevant ICUs.

## 1 Introduction

With improving long‐term survival among critically ill patients, understanding the short‐ and long‐term impact of intensive care on health‐related quality of life (HRQoL) has become increasingly important. Despite advances in critical care, many patients experience persistent physical, neurocognitive and psychological impairments, commonly referred to as post‐intensive care syndrome (PICS), which prolong recovery and hinder return to work, daily activities and social participation [1, 2, 3]. An expanding body of literature demonstrates that PICS has detrimental consequences for patients and their families, increasing healthcare resource utilisation and, in turn, placing additional burdens on the broader socioeconomic system [4].

Follow‐up of ICU survivors after discharge has been recommended since 2009 [5], with increasing emphasis on early identification of patients at risk for PICS and referral for identified problems [6]. However, current guidelines provide limited guidance on how ICU follow‐up should be organised, delivered or evaluated [2, 7, 8]. Uncertainty remains regarding effectiveness and cost‐efficiency [6, 7, 9], largely due to a lack of randomised controlled trials, and methodological variation across studies [6, 10, 11]. Evidence from qualitative studies suggests that ICU follow‐up may support psychological recovery [12, 13, 14], is valued by patients [12, 14] and provides a forum for addressing concerns related to the ICU experience [12, 13, 14, 15, 16]. However, these findings are based on low‐certainty evidence and do not establish effectiveness. Despite these uncertainties, ICU follow‐up has been implemented in many countries [6, 11]. In practice, ICU follow‐up varies considerably with respect to patient selection, timing and content, reflecting the absence of consensus and contributing to inconsistent care [17].

Adherence to follow‐up remains low, with only a minority of eligible patients participating [16] and barriers such as limited resources, staffing constraints and challenges in identifying patients most likely to benefit being common [18]. Most programmes are nurse‐led, sometimes involving multidisciplinary teams and typically include face‐to‐face or telephone consultations with assessments of physical and psychological status [2, 17].

In Sweden, participation in ICU follow‐up is incomplete and influenced by patient characteristics. A Swedish registry study reported that only 57% of patients attended follow‐up, indicating substantial attrition and potential selection bias [19]. In addition, systematic screening is not yet routinely implemented, and access to a multiprofessional team is inconsistent [17], contributing to many patients with persistent impairments remaining unidentified and unsupported. However, when the available evidence is weak, further research is needed to reduce uncertainty.

Therefore, given the paucity of high‐quality evidence in this area, we developed a consensus recommendation, rather than an evidence‐based guideline, to inform the organisation, delivery and content of ICU follow‐up for adult ICU survivors in Sweden. It addresses patient selection, timing, assessment and care pathways for managing PICS, aiming to support the continuity of care, identify high‐risk patients and provide a framework to guide resource use and patient outcomes.

## 2 Methods

### 2.1 The Development Process

This consensus recommendation was developed on the initiative of the National System for Knowledge‐driven healthcare management (NPO) [20], which commissioned the creation of regional and national guidelines for the follow‐up of critically ill adults [20]. A multidisciplinary task force with experts in ICU follow‐up was formed to develop the recommendation. The development process followed a predefined evidence‐based practice model [21] (Figure 1), structured as a stepwise progression from formulation of clinical questions, to identifying the best evidence to answer the questions, to appraising the evidence for validity and usefulness, to integrating it with experts and stakeholder engagement and to apply the results in clinical recommendations [21]. These clinical questions were prioritised because they concern the fundamental components of ICU follow‐up that influence patient outcomes and healthcare resource utilisation. Decisions regarding patient selection, timing, organisation and assessment determine the reach, content and delivery of follow‐up and therefore underpin the effectiveness of any subsequent interventions. ‘Which patients should be followed up’, ‘At what time‐point should patients be followed up’, ‘How should follow‐up be organised’ and ‘What problems should be addressed’.

**FIGURE 1:**
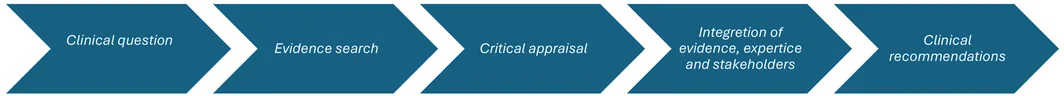
Overview of the evidence practice model used for guideline development.

The quality of the consensus recommendation was reported using the Appraisal of Guidelines for Research and Evaluation (AGREE II) instrument, a validated tool designed to assess the methodological rigour and transparency of guideline development. The recommendation was evaluated across the six AGREE II domains according to the instrument's standardised scoring system [22] (Supplementary File 1).

A multidisciplinary working group in the Stockholm region was convened to ensure broad clinical relevance and representation from key professional groups involved in ICU follow‐up. Each ICU nominated health‐care professionals with clinical experience and responsibilities in post‐ICU follow‐up care to represent their unit. The group consisted of critical care nurses (*n* = 6), physicians (*n* = 3), physiotherapists (*n* = 3) and occupational therapists (*n* = 1) from six ICUs. Clinical experience was defined as ongoing involvement in the assessment and follow‐up of former ICU patients. No predefined number of years' experience was required.

The team worked collaboratively throughout the process, while assigning professional‐specific tasks to ensure that each discipline's perspective was represented. To facilitate this, each professional discipline formed a dedicated subgroup. Drawing on their specific clinical expertise, professional experience and available scientific evidence, these subgroups developed proposals and drafted content for their respective areas of competence. These drafts were subsequently reviewed and discussed by the entire task force group to ensure coherence, relevance and consistency across the draft.

The working group participated in iterative rounds of discussion, revision and consensus‐building to refine the recommendations. Divergent viewpoints were explored collaboratively, and consensus was achieved through structured deliberation.

### References

2.2

A comprehensive list of relevant keywords was initially developed, to capture a wide range of factors for follow‐up within PICS (physical, psychological and cognitive dysfunction). A professional librarian refined and structured the search strategy using PICO (Table 1) and the terms ‘intensive care’, ‘critical care’, ‘follow‐up’, ‘physical’, ‘psychological’ and ‘cognitive’ (Supplementary File 2). Four databases were included in all searches: PubMed, MEDLINE, Cochrane Database of Systematic Reviews and Scopus. The scope of the search encompassed short‐ and long‐term follow‐up of adult medical, surgical and trauma patients treated in the ICU. The searches were limited to English articles involving adult patients (≥ 18 years), published between 1 January 2007, and 31 December 2024. Studies focusing on paediatric or burns ICUs were excluded, as these populations follow separate routines for follow‐up and were not addressed in this search.

**TABLE 1:** The PICO summary table.

Population	Adult ICU survivors across medical, surgical and trauma specialties
Intervention	Any structured ICU follow‐up initiative, including outpatient clinics, ward visits, screening procedures and follow‐up programmes
Comparator	
Outcomes	Identification and management of PICS‐related physical, cognitive or psychological problems, patient‐reported outcomes, recovery trajectories, organisation of follow‐up services

Three researchers (A.S., L.O. and M.R.) conducted the first and secondary screening. Titles and abstracts were screened, and studies that did not address patient selection, timing, organisation, or content of ICU follow‐up were excluded (Figure 2). Thirteen articles met the inclusion criteria and were retrieved for secondary screening with full‐text reviews; nine were excluded due to not being systematic reviews, and four were eventually included (Supplementary File 3). The three researchers (A.S., L.O. and M.R.) independently appraised the methodological quality of the included studies using the Critical Appraisal Skills Program (CASP) Checklist (www.casp‐uk.net) (Supplementary File 4). Results were then compared in a consensus meeting. Each CASP item was scored as met, unclear, or not met. The overall evidence level was assigned according to the following criteria: studies that met the majority of CASP criteria were considered of high quality; studies meeting several CASP criteria but containing methodological weaknesses were regarded as being of moderate quality; and studies with substantial methodological shortcomings were of low quality. Studies with low quality or studies where methodological validity could not be established were excluded. Data extraction was performed independently by all members of the research team using a predefined data abstraction framework. Extracted data were then compared across reviewers, and any discrepancies were discussed and resolved through consensus to ensure accuracy and consistency. A narrative synthesis was conducted based on the extracted data. Each included study was read and re‐read in full, and relevant findings were systematically examined to identify recurring concepts, patterns and relationships across studies. These patterns were iteratively refined and integrated to develop a coherent synthesis of the evidence.

**FIGURE 2:**
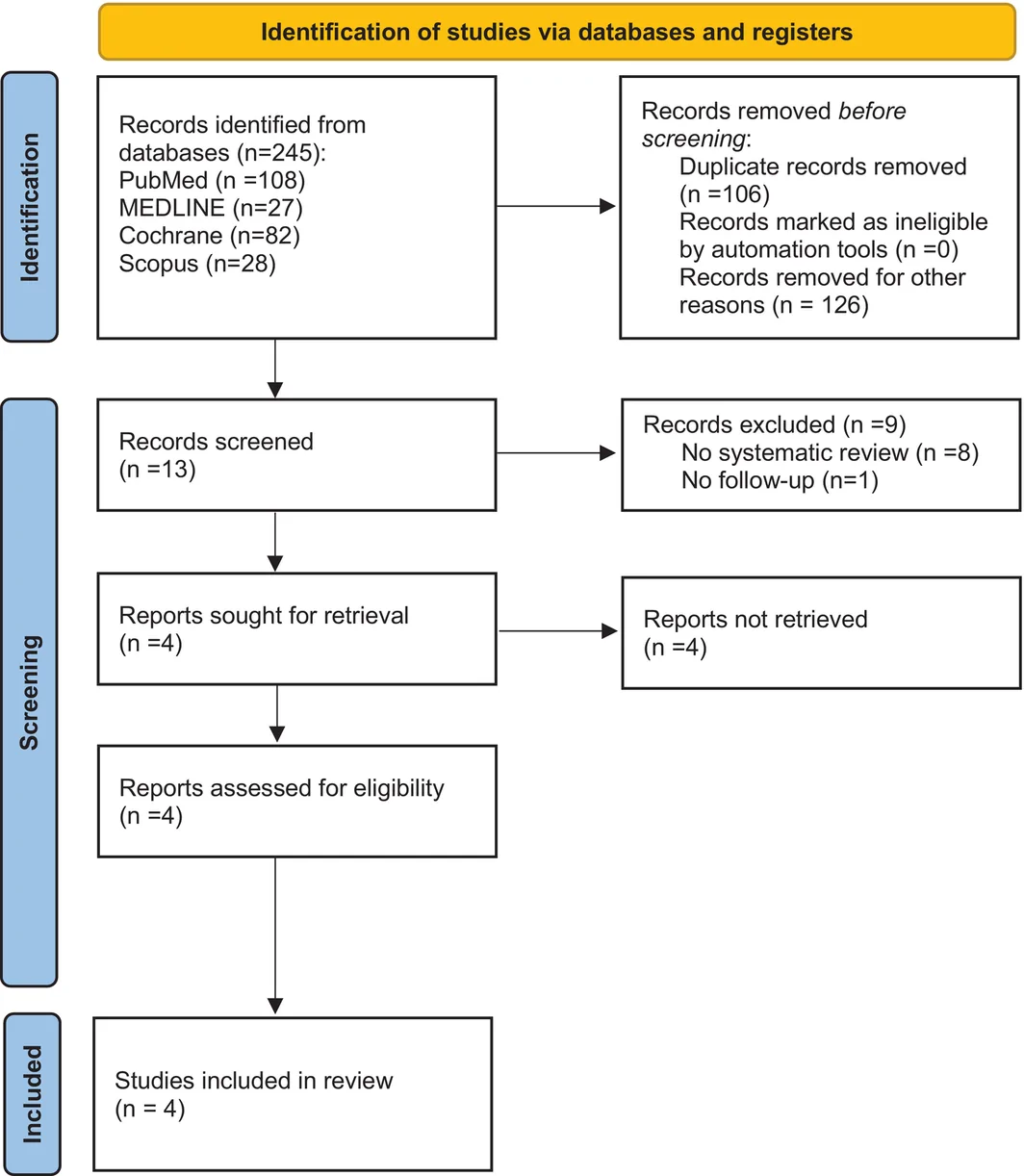
PRISMA 2020 flow diagram for new systematic reviews which included searches of databases and registers only. *Source:* Page et al. [23].

The development team collectively reviewed the evidence within each domain. In a series of meetings, the working groups created actionable recommendations. Internal review and feedback on the evidence review were undertaken to ensure accuracy of content and consistency. Relevant domains of the AGREE II instrument were used to guide the evidence review process and support methodological rigour [22].

### 2.3 Stakeholder and Patient Involvement

To ensure that the recommendation reflected the needs and experiences of clinical users, several stakeholder engagement activities were conducted. First, a dedicated workshop for critical care nurses was held in conjunction with the annual conference of the Swedish Association of Nurse Anesthetists and Critical Care Nurses (ANIVA). Thirty critical care nurses from across Sweden participated. During facilitated group discussions, participants shared their experiences and views on key aspects of ICU follow‐up, including criteria for patient selection, optimal timing of follow‐up and the organisation and content of follow‐up services.

Second, a seminar for clinicians (critical care nurses, physicians, assistant nurses, physical therapists) involved in ICU follow‐up was held at the yearly conference, arranged by The Swedish Intensive Care Quality Register (SIR). At this meeting, results from the literature synthesis and the ANIVA workshop were presented to the 74 attendees, followed by structured small‐group discussions. Participants reflected on the findings and contributed their own experiences with ICU follow‐up programmes.

Third, patient perspectives were incorporated through a structured group discussion with four patient representatives who participated in a digital meeting. During this meeting, the draft was presented, and participants were invited to comment on its content, structure and proposed recommendations.

Following the review by stakeholders, clinical users and patient representatives, the draft recommendation underwent further refinement. Feedback from all groups was carefully considered and discussed within the task force to ensure that the recommendations were informed by the available evidence, clinically relevant and feasible to implement and aligned with patient needs and preferences.

### 2.4 External Review

The final draft of the recommendation underwent extensive external peer review by representatives from 12 of the Swedish regions, professional societies for anaesthesia and critical care nurses and physicians, the Swedish Society for occupational and physiotherapists, the Swedish Intensive Care Registry, the E‐health agency and NPO for rehabilitation, habilitation and insurance medicine. All comments were compiled and evaluated by the task force group, after which a revised version of the recommendation was circulated and formally approved.

## 3 Results

### 3.1 Literature Review

The consensus recommendations are based on available evidence published up to December 2024. The search yielded 139 unique references. Four reviews met the inclusion criteria, comprising three systematic reviews and one integrative review (Table 2). The reviews evaluated ICU follow‐up interventions, targeting physical, psychological, cognitive and HRQoL after critical illness. Findings were heterogeneous, with some studies reporting benefits in identification of rehabilitation needs and patient‐reported outcomes, while others found limited effects. The overall certainty of evidence was considered low to moderate due to heterogeneity in interventions, outcomes and designs. In cases where evidence was insufficient or consensus could not be reached, no recommendation was issued. These findings were considered together with clinical expertise and feasibility when formulating the recommendations. The consensus recommendation is presented in Figure 3.

**TABLE 2:** Reviewed and included articles.

References	Design/method	Outcome	Results	Level of evidence
Josepha op't Hoog et al. [24]	Systematic review and meta‐analysis in six databases. Non‐randomised controlled trials, RCT, stepped‐wedge studies, interrupted time series analysis and before after studies. 983 participants. Patients and/or family members ≥ 18 years.	Outcomes: PICS related to physical, cognitive or psychological symptoms.	Five studies included two RCT, one a block intervention study, one a pre‐test–post‐test control group design and one non‐RCT. The studies show no significant differences in favour of transitional care interventions on physical or psychological aspects of PICS‐(F). One study with a nurse‐led structured follow‐up programme showed a significant difference in physical function at 3 months.	Quality among the studies differs from moderate to high. There was a substantial clinical heterogeneity that made pooling for primary outcomes unfeasible.
Schofield‐Robinson et al. [25]	Systematic review, search in four databases, clinical trial registers for ongoing studies and grey literature. RCT and non‐RCT comparing effect of follow‐up using a structured programme and no follow‐up service or standard care. 1707 participants. Patients ≥ 18 years cared in a Level 3 ICU.	Outcomes: health‐related quality of life, anxiety and depression, post‐traumatic stress disorder (PTSD) or physical and mental function, mortality and return to work.	Five studies included four RCT and one non‐RCT. There was little or no difference in the outcomes between participants who received a follow‐up service and participants who received standard care.	There was insufficient evidence, from a limited number of studies, to determine whether ICU follow‐up services are effective in identifying and addressing the unmet health needs of ICU survivors.
Jonasdottir et al. [10]	Integrative review, search in four databases. Empirical qualitative and quantitative studies and nonempirical literature reflecting service improvement. Studies with follow‐up reported as an intervention or a service within a hospital or a hospital clinic. 8767 participants. Patients ≥ 18 years.	Outcomes: Structure and content in follow‐up and the types of variables used.	Seventeen studies included, two RCT, a case–control study, four before‐and‐after studies, a qualitative study and nine descriptive and evaluative studies. Three models of nurse‐led follow‐up were identified: ward visits, follow‐up clinics and telephone follow‐up. Interventions ranged from clinical assessment to psychosocial support. Positive effects were mainly reported for patient satisfaction up to 6 months post discharge. Follow‐up might promote patients' health and enable use of adequate resources.	The weaknesses of the study was the heterogeneity of the included studies, deficiencies in study methodology and the low number of studies.
Jensen [26]	Systematic literature review, search in five databases. RCT studies investigating post‐ICU consultations in adults with post ICU follow‐up compared with control which does not provide any posticum consultation. 855 participants. Medical and surgical patients ≥ 18 years.	Outcomes: quality of life (QOL), anxiety, depression, posttraumatic stress disorder (PTSD), physical ability, cognitive function and return to work.	Three multicentre studies, two single centres. One study conducted in Australia, one in USA, one in six European countries and two in UK. Follow‐up consultations might reduce symptoms of PTSD by 3–6 months after ICU discharge in ICU survivors, but without affecting HRQoL and other outcomes investigated.	The overall quality of evidence for effect of post‐ICU follow‐up consultations was low.

**FIGURE 3:**
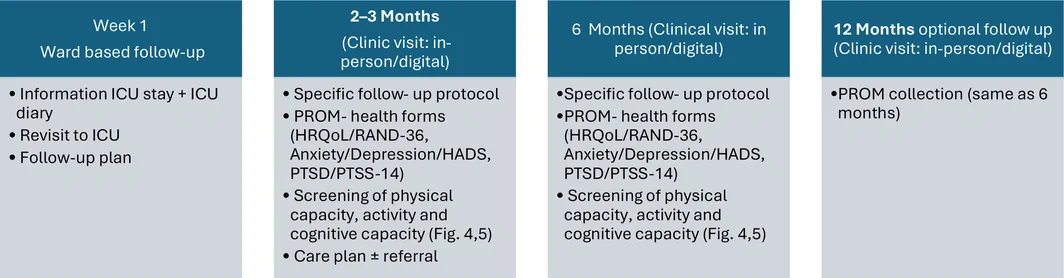
Recommendation for Follow‐up after intensive care. HADS, Hospital Anxiety and Depression Scale; HRQoL, Health Related Quality of Life; PTSS, post‐traumatic stress symptoms; RAND‐36, Short Form Health Survey.

### 3.2 Stakeholder and Patient Involvement

During the national seminar for clinicians, they highlighted the need for a more standardised approach to follow‐up, both in terms of organisational structure and content. Stakeholders highlighted that standardisation could clarify clinical responsibilities, support equitable provision of follow‐up services and facilitate the identification of resource needs. They also emphasised that greater consistency across follow‐up practices would help reveal knowledge gaps in the care of ICU survivors along the recovery trajectory. However, they had no practical implications for the draft recommendation and no structure was changed.

Patient representatives supported the recommendations and emphasised the particular importance of the ward visit as part of ICU follow‐up. They described this visit as a valuable opportunity for processing their ICU experiences and potentially reducing later stress‐related symptoms. The patient representatives considered the number of screening questionnaires included in the follow‐up acceptable when their purpose and clinical relevance were clearly explained. These insights were incorporated into the final recommendations by reinforcing the value of the ward visit as a core component of follow‐up and by ensuring that the use of screening tools is justified, purposeful and clearly explained to patients.

### 3.3 External Review

The external stakeholders' review resulted in the structured assessment forms for screening physical function, activity in daily life and cognitive function follow‐up being added to support identification of rehabilitation needs. The external review also provided feedback regarding the inclusion of additional screening of physical function and limitations in activities of daily living, and emphasised that the ICU follow‐up should be provided by a multiprofessional team due to the multifaceted nature of the PICS problems.

### 3.4 From Evidence to Recommendations

Evidence supporting ICU follow‐up was generally of low certainty. Nevertheless, the task force judged that the potential benefit of identifying unmet rehabilitation needs, the low risk of harm, strong patient preferences and the importance of equitable access to follow‐up services justified issuing this consensus recommendation.

#### 3.4.1 Consensus Recommendation

##### 3.4.1.1 Selection of Patients for Follow‐Up

*Key clinical question*: *Which patients should be followed up*?

*We recommend that clinicians consider inviting* all adult patients treated in an ICU for ≥ 48 h to structured ICU follow‐up.

The reviewed literature provided little guidance regarding patient selection for ICU follow‐up services. Across the four reviews, no consensus exists regarding inclusion criteria, and no specific ICU length‐of‐stay cutoff is consistently recommended [10, 24, 25, 26]. Eligibility criteria varied substantially among studies and were typically based on local models for follow‐up rather than standardised indicators.

In the absence of evidence, adopting a pragmatic threshold of > 48 h in the ICU helps to ensure that individuals who develop new or worsening physical, cognitive, or psychological problems after critical illness are not inadvertently excluded from routine follow‐up. This approach may result in a larger cohort of patients being offered follow‐up, but it increases the likelihood of identifying patients who would benefit from post‐ICU support. Therefore, this is a conditional recommendation since it is based on limited evidence.

##### 3.4.1.2 Time Points for Follow‐Up

*Key clinical question*: At what time points should patients be followed up?

*We recommend that clinicians consider* offering all ICU survivors an initial follow‐up contact within the first week after ICU discharge, ideally on the ward, to assess clinical status, identify early problems and ensure continuity of care. We further recommend that patients and their families be offered an outpatient follow‐up visit at 2–3 months after ICU discharge, with the option of an additional visit within 6 months if clinically indicated.

There is moderate‐quality evidence regarding interventions delivered at 2–3 months after ICU discharge, but not necessarily for the timing of follow‐up itself. In the literature review, the interventions varied regarding time to follow‐up and number of consultations [10, 25, 26]. One of the four reviews included suggested that follow‐up should be initiated within the first week after ICU discharge. This recommendation was based on the potential benefits of early follow‐up in supporting the transition from ICU to ward care, identifying patient needs and complications at an early stage, and facilitating communication and collaboration between ICU staff, ward teams, patients and their families [10]. Outpatient evaluation at 2–3 months aligns with common practice patterns and helps address ongoing physical, cognitive and psychological recovery needs [10, 25, 26]. A second visit within 6 months allows timely reassessment and referral to appropriate multidisciplinary or primary care services when needed [10]. These follow‐ups may be conducted either in person or digitally, depending on feasibility and patient preference. Furthermore, responses from the questionnaires collected can serve as a basis for discussion of needs prior to each visit.

##### 3.4.1.3 Organisation and Content of Follow‐Up

*Key clinical question*: How should follow‐up be organised and what problems should be addressed?

*We recommend* that outpatient ICU follow‐up be led by clinical experts in intensive care and, where possible, delivered by a multidisciplinary team (e.g., ICU nurses, physicians, physiotherapists) to ensure comprehensive assessment and management of post‐ICU problems.

*We recommend* that follow‐up consultations include the provision of information about the ICU stay and treatment and allow time for patients and families to ask questions and receive explanations about symptoms commonly experienced after critical illness.

*We recommend* that follow‐up consultations include a structured assessment to identify symptoms of post‐traumatic stress, anxiety, depression, cognitive impairment, reduced physical function, limitations in activities of daily living, HRQoL and unmet rehabilitation needs. This assessment may be conducted using validated instruments where available, together with clinical interviews and structured screening questions regarding symptoms, daily functioning, perceived recovery and rehabilitation need. Patients with identified physical, psychological, or cognitive problems should be referred to the appropriate healthcare provider or specialist service for further evaluation and management.

In the literature follow‐up consultations aimed to assess patients' recovery and identify problems that delayed recovery [10]. Consultations commonly included assessment of psychological, cognitive, physical recovery and HRQoL. Several studies used validated instruments and objective tests, including PTSS‐14, HADS, the Montreal Cognitive Assessment (MoCA), the Cognitive Failures Questionnaire (CFQ), handgrip strength, six‐minute walk test, Timed Up and Go test and RAND‐36, to support the identification of patients who need further interventions [10, 25, 26].

Given the limited and heterogeneous evidence base, this consensus recommendation should be interpreted as pragmatic recommendations informed by the available literature and expert clinical judgement.

## 4 Discussion

This Swedish consensus recommendation proposes a structured, standardised approach to follow‐up after critical illness to improve identification and management of PICS. It suggests that all adults treated in the ICU for ≥ 48 h receive early and ongoing follow‐up, beginning with contact within the first week and continuing with multidisciplinary outpatient assessment at 2–3 months. Emphasis is placed on using validated screening tools, providing information to patients and families, and ensuring referral to appropriate services when physical, psychological, or cognitive problems are identified.

Our consensus recommendations align with existing international guidelines and consensus statements, while also extending them in some important ways. Consistent with the British National Institute for Health and Care Excellence guideline (CG83) [5], we emphasise structured follow‐up after critical illness, including a multidisciplinary assessment at 2–3 months to identify physical, cognitive and psychological problems and to coordinate ongoing care. Similarly, our recommendation for systematic screening across these domains is in line with the Society of Critical Care Medicine consensus, which advocates assessments after hospital discharge using validated tools and highlights the importance of identifying patients at risk for long‐term impairments [27]. In addition, our focus on comprehensive assessment and referral pathways corresponds with the multimodal rehabilitation guideline for PICS coordinated by the German Society for Neurorehabilitation, which also underlines the need for repeated evaluation and coordinated, multidisciplinary management of physical, cognitive and psychological sequelae [28]. However, our consensus recommendation provides greater operational clarity by outlining a more explicit follow‐up pathway, including early contact within the first week after ICU discharge followed by structured outpatient follow‐up. By specifying timing and referral strategies, it extends beyond existing recommendations. We acknowledge that implementation of these recommendations may require additional healthcare resources. Nevertheless, we consider this is an important area for prioritisation, given its potential benefits for patients, healthcare systems and society at large. Furthermore, a more structured and standardised follow‐up model could facilitate research and enable robust evaluation of the effectiveness, feasibility and resource implications of post‐ICU follow‐up care.

### 4.1 Implementation and Implications for Clinical Practice

Implementation of structured ICU follow‐up requires substantial organisational and workforce resources. A multidisciplinary approach is essential, involving ICU clinicians alongside physiotherapists, occupational therapists and other rehabilitation professionals. However, staffing constraints and competing clinical priorities may limit the feasibility of establishing dedicated follow‐up services in many settings.

The consensus recommendation adopts a pragmatic approach to enhance feasibility, using ICU length of stay as a simple and applicable criterion for identifying patients at risk, despite its limitations as a predictor of long‐term outcomes [29, 30, 31]. While more complex risk stratification models may offer greater precision, their routine use is currently limited by feasibility, validation and clinical usability [30, 31, 32]. ICU length of stay was therefore selected as a pragmatic compromise, enabling consistent identification of at‐risk patients in everyday practice, and should be interpreted alongside clinical judgement and individual patient factors.

To support implementation, follow‐up may be integrated into existing care pathways, with emphasis on protected time for assessments and effective coordination across ICU, ward and community services. Robust documentation and communication systems are needed to maintain continuity of care and ensure consistent application of rehabilitation plans throughout the patient's journey. Given the resource implications and variability in local infrastructure, the recommended model should be viewed as a flexible framework rather than a rigid standard. A stepwise approach, for example, prioritising patients at highest risk or gradually expanding services, may facilitate more achievable implementation. Institutional support, clear policies and systems for outcome monitoring are key enablers of sustainable practice.

Although developed in Sweden, these principles may be applicable across healthcare systems and offer a generalisable approach to improving follow‐up care after critical illness.

### 4.2 Strengths and Limitations

A major strength is the consensus recommendation's structured and pragmatic framework, integrating validated assessments, defined follow‐up and multidisciplinary referral pathways to support consistent post‐ICU care. However, some limitations should be acknowledged. First, the evidence base underpinning follow‐up interventions after critical illness remains heterogeneous and, in some areas, limited, particularly regarding patient selection, optimal timing and long‐term impact of follow‐up services. As a result, some recommendations are necessarily based on expert consensus in addition to the available literature. Second, while the recommendation aims to be pragmatic, its implementation may be challenging in routine clinical practice. In particular, the establishment of structured follow‐up services requires sufficient staffing, time and organisational resources, which may not be readily available in all settings. Variability in local infrastructure, access to multidisciplinary teams and availability of specialist services may further influence the ability to deliver the recommended pathway. A further limitation relates to the literature base underpinning the recommendation. The evidence synthesis primarily relied on existing systematic reviews rather than conducting new independent searches for individual studies. An integrative review was included because it provided a broader understanding of patients' symptoms, needs and experiences following critical illness across different study designs. This review was used primarily to inform the identification of relevant follow‐up domains and patient needs, rather than as evidence of intervention efficacy.

Also, the total number of participants reported across systematic reviews should be interpreted with caution, as not all participants contributed to each review question. Some primary studies are included in more than one review, resulting in overlap. The CASP checklist evaluates the methodological quality of the systematic reviews rather than the quality of the individual primary studies. While this approach allows for an efficient and comprehensive overview of the available evidence, it also means that the recommendation reflects the heterogeneity and methodological limitations present in the underlying reviews, including variation in intervention components, patient populations and outcomes. In addition, more recent primary studies not yet included in published reviews may have been missed. This approach may limit both the level of detail and the timeliness of the evidence informing the recommendations. Finally, although cost considerations informed the development process, a formal cost or cost‐effectiveness analysis was beyond the scope of this work. Future studies should evaluate the economic impact and sustainability of different follow‐up models.

### 4.3 Future Directions

Further research is needed to evaluate the feasibility, effectiveness and resource utilisation of structured follow‐up models and rehabilitation pathways in real‐world clinical settings, including their impact on patient outcomes and healthcare utilisation. Studies should also determine the optimal timing, frequency and content of follow‐up assessments, alongside formal cost‐effectiveness analysis to inform policy and resource allocation. In addition, there is a need to explore how follow‐up services can be organised within healthcare systems. One potential approach is to develop hospital‐wide services independent of individual ICUs, supported by dedicated multidisciplinary teams, which may facilitate coordination and scalability. The economic impact, sustainability and scalability of such models require further investigation. Finally, patient‐centred research is essential to better understand survivors' experiences and priorities, ensuring that future guideline development is informed by both clinical evidence and lived experience. Although considerations related to cost and resource availability informed the consensus recommendation development process, these were not formally evaluated, highlighting an important area for future study.

## 5 Conclusion

The consensus recommendation should be regarded as a pragmatic model for ICU follow‐up, emphasising early contact, multidisciplinary involvement and structured assessment using validated tools. By targeting both short‐ and longer‐term needs, it facilitates the timely identification and management of physical, psychological and cognitive sequelae after critical illness.

Although based on low‐certainty evidence, several recommendations were considered strong recommendations because of the high burden of unmet patient needs, the low likelihood of harm, the lack of alternative care pathways and the consistent preferences expressed by patients and healthcare professionals.

Implementation of such an approach has the potential to support the continuity of care, guide resource use and promote more equitable and consistent follow‐up for ICU survivors and their families. Although developed in Sweden, these principles may apply to other health systems, offering a pathway to improve long‐term outcomes for ICU survivors.

## Author Contributions

E.Å., A.S., M.R., O.D., and L.O. contributed to conception and design, or acquisition of data, or analysis and interpretation of data. E.Å., A.S., M.R., and L.O. drafted the manuscript. All authors revised the draft and approved the final version.

## Funding

The authors have nothing to report.

## Ethics Statement

The authors have nothing to report.

## Conflicts of Interest

The authors declare no conflicts of interest.

## Supporting information


**Supplementary File 1.** Agree‐reporting Guideline checklist.

**Supplementary File 2.** Search string Pubmed.

**Supplementary File 3.** Reviewed articles.

**Supplementay File 4.** CASP—Methodological quality appraisal of each included study.

## Data Availability

The data that support the findings of this study are available from the corresponding author upon reasonable request.

## References

[1] S. Rai , L. Anthony , D. M. Needham , et al., “Barriers to Rehabilitation After Critical Illness: A Survey of Multidisciplinary Healthcare Professionals Caring for ICU Survivors in an Acute Care Hospital,” Australian Critical Care 33, no. 3 (2020): 264–271, 10.1016/j.aucc.2019.05.006. 31402265

[2] A. F. Rousseau , H. C. Prescott , S. J. Brett , et al., “Long‐Term Outcomes After Critical Illness: Recent Insights,” Critical Care 25, no. 1 (2021): 108, 10.1186/s13054-021-03535-3. PMC7968190 33731201

[3] D. M. Needham , J. Davidson , H. Cohen , et al., “Improving Long‐Term Outcomes After Discharge From Intensive Care Unit: Report From a Stakeholders' Conference,” Critical Care Medicine 40, no. 2 (2012): 502–509, 10.1097/CCM.0b013e318232da75. 21946660

[4] E. L. Hirshberg , E. L. Wilson , V. Stanfield , et al., “Impact of Critical Illness on Resource Utilization: A Comparison of Use in the Year Before and After ICU Admission,” Critical Care Medicine 47 (2019): 1497–1504, 10.1097/CCM.0000000000003970. PMC6798747 31517693

[5] National Institute for Health and Care Excellence , Rehabilitation After Critical Illness in Adults (CG83) (NICE, 2009), https://www.nice.org.uk/guidance/cg83. 39480975

[6] S. Lasiter , S. K. Oles , J. Mundell , S. London , and B. Khan , “Critical Care Follow‐Up Clinics: A Scoping Review of Interventions and Outcomes,” Clinical Nurse Specialist 30, no. 4 (2016): 227–237, 10.1097/NUR.0000000000000219. PMC4911825 27309787

[7] B. H. Cuthbertson , J. Rattray , M. K. Campbell , et al., “The PRaCTICaL Study of Nurse Led, Intensive Care Follow‐Up Programmes for Improving Long Term Outcomes From Critical Illness: A Pragmatic Randomised Controlled Trial,” BMJ 339 (2009): b3723, 10.1136/bmj.b3723. PMC2763078 19837741

[8] J. F. Jensen , I. Egerod , M. H. Bestle , et al., “A Recovery Program to Improve Quality of Life, Sense of Coherence and Psychological Health in ICU Survivors: A Multicenter Randomized Controlled Trial, the RAPIT Study,” Intensive Care Medicine 42, no. 11 (2016): 1733–1743, 10.1007/s00134-016-4522-1. 27695894

[9] C. Jones , “Recovery Post ICU,” Intensive & Critical Care Nursing 30, no. 5 (2014): 239–245, 10.1016/j.iccn.2014.06.001. 25065538

[10] R. J. Jonasdottir , M. E. Klinke , and H. Jonsdottir , “Integrative Review of Nurse‐Led Follow‐Up After Discharge From the ICU,” Journal of Clinical Nursing 25, no. 1–2 (2016): 20–37, 10.1111/jocn.12939. 26420520

[11] K. P. Mayer , H. Boustany , E. P. Cassity , et al., “ICU Recovery Clinic Attendance, Attrition, and Patient Outcomes: The Impact of Severity of Illness, Gender, and Rurality,” Critical Care Explorations 2, no. 10 (2020): e0206, 10.1097/CCE.0000000000000206. PMC7523871 33063022

[12] K. J. Farley , G. M. Eastwood , and R. Bellomo , “A Feasibility Study of Functional Status and Follow‐Up Clinic Preferences of Patients at High Risk of Post Intensive Care Syndrome,” Anaesthesia and Intensive Care 44, no. 3 (2016): 413–419, 10.1177/0310057X1604400310. 27246943

[13] J. F. Jensen , D. Overgaard , M. H. Bestle , D. F. Christensen , J. Rattray , and I. Egerod , “Intervention Fidelity in Postintensive Care Follow‐Up Consultations at Ten Sites in the RAPIT‐Trial: A Mixed‐Methods Evaluation,” Journal of Advanced Nursing 75, no. 4 (2019): 862–875, 10.1111/jan.13949. 30644124

[14] C. Robinson , E. Hibbert , A. J. Bastin , et al., “An International Study Exploring the Experience of Survivors of Critical Illness as Volunteers Within ICU Recovery Services,” Critical Care Explorations 2, no. 11 (2020): e0273, 10.1097/CCE.0000000000000273. PMC8191690 34124686

[15] L. Denehy and D. Elliott , “Strategies for Post ICU Rehabilitation,” Current Opinion in Critical Care 18, no. 5 (2012): 503–508, 10.1097/MCC.0b013e328357f064. 22914429

[16] A. R. Schandl , O. R. Brattstrom , A. Svensson‐Raskh , E. M. Hellgren , M. D. Falkenhav , and P. V. Sackey , “Screening and Treatment of Problems After Intensive Care: A Descriptive Study of Multidisciplinary Follow‐Up,” Intensive & Critical Care Nursing 27, no. 2 (2011): 94–101, 10.1016/j.iccn.2011.01.006. 21334207

[17] N. Nakanishi , K. Liu , J. Hatakeyama , et al., “Post‐Intensive Care Syndrome Follow‐Up System After Hospital Discharge: A Narrative Review,” Journal of Intensive Care 12, no. 1 (2024): 2, 10.1186/s40560-023-00716-w. PMC10785368 38217059

[18] K. J. Haines , J. McPeake , E. Hibbert , L. M. Boehm , K. Aparanji , and R. N. Bakhru , “Enablers and Barriers to Implementing ICU Follow‐Up Clinics and Peer Support Groups Following Critical Illness: The Thrive Collaboratives,” Critical Care Medicine 47, no. 9 (2019): 1194–1200, 10.1097/CCM.0000000000003818. PMC6699486 31241499

[19] C. Glimelius Petersson , L. Jakobsson , A. Westergren , and I. Bergbom , “Factors and Health‐Related Quality of Life Associated With Participation in a Post‐ICU Follow‐Up. A Register Study,” Acta Anaesthesiologica Scandinavica 65, no. 7 (2021): 902–911, 10.1111/aas.13811. 33650105

[20] National System for Knowledge‐Driven Management Within Swedish Healthcare , “Riktlinje för uppföljning av vuxna som vårdats på intensivvårdsavdelwning,” 202, 2026.

[21] D. L. Sackett , “Evidence‐Based Medicine,” Seminars in Perinatology 21 (1997): 3–5, 10.1016/s0146-0005(97)80013-4. 9190027

[22] M. C. Brouwers , K. Kerkvliet , K. Spithoff , and AGREE Next Steps Consortium , “The AGREE Reporting Checklist: A Tool to Improve Reporting of Clinical Practice Guidelines,” BMJ 352 (2016): i1152. 10.1136/bmj.i1152 PMC5118873 26957104

[23] M. J. Page , J. E. McKenzie , P. M. Bossuyt , et al., “The PRISMA 2020 Statement: An Updated Guideline for Reporting Systematic Reviews,” BMJ 372 (2020): n71, 10.1136/bmj.n71. PMC8005924 33782057

[24] S. A. Johanna Josepha Op't Hoog , A. M. Eskes , M. P. Johanna van Mersbergen‐de Bruin , et al., “The Effects of Intensive Care Unit‐Initiated Transitional Care Interventions on Elements of Post‐Intensive Care Syndrome: A Systematic Review and Meta‐Analysis,” Australian Critical Care 35, no. 3 (2022): 309–320, 10.1016/j.aucc.2021.04.010. 34120805

[25] O. J. Schofield‐Robinson , S. R. Lewis , A. F. Smith , J. McPeake , and P. Alderson , “Follow‐Up Services for Improving Long‐Term Outcomes in Intensive Care Unit (ICU) Survivors,” Cochrane Database of Systematic Reviews 11 (2018): CD012701, 10.1002/14651858.CD012701.pub2. PMC6517170 30388297

[26] J. F. Jensen , T. Thomsen , D. Overgaard , M. H. Bestle , D. Christensen , and I. Egerod , “Impact of Follow‐Up Consultations for ICU Survivors on Post‐ICU Syndrome: A Systematic Review and Meta‐Analysis,” Intensive Care Medicine 41, no. 5 (2015): 763–775, 10.1007/s00134-015-3689-1. 25731633

[27] M. E. Mikkelsen , M. Still , B. J. Anderson , et al., “Society of Critical Care Medicine's International Consensus Conference on Prediction and Identification of Long‐Term Impairments After Critical Illness,” Critical Care Medicine 48, no. 11 (2020): 1670–1679, 10.1097/CCM.0000000000004586. 32947467

[28] C. Renner , M. M. Jeitziner , M. Albert , et al., “Guideline on Multimodal Rehabilitation for Patients With Post‐Intensive Care Syndrome,” Critical Care 27, no. 1 (2023): 301, 10.1186/s13054-023-04569-5. PMC10392009 37525219

[29] L. Orwelius , A. Nordlund , P. Nordlund , et al., “Pre‐Existing Disease: The Most Important Factor for Health Related Quality of Life Long‐Term After Critical Illness: A Prospective, Longitudinal, Multicentre Trial,” Critical Care 14, no. 2 (2010): R67, 10.1186/cc8967. PMC2887189 20398310

[30] A. Milton , A. Schandl , I. W. Soliman , et al., “Development of an ICU Discharge Instrument Predicting Psychological Morbidity: A Multinational Study,” Intensive Care Medicine 44, no. 12 (2018): 2038–2047, 10.1007/s00134-018-5467-3. PMC6280826 30467678

[31] A. Milton , A. Schandl , I. Soliman , et al., “ICU Discharge Screening for Prediction of New‐Onset Physical Disability‐A Multinational Cohort Study,” Acta Anaesthesiologica Scandinavica 64, no. 6 (2020): 789–797, 10.1111/aas.13563. 32083323

[32] J. K. Pilowsky , A. von Huben , R. Elliott , and M. A. Roche , “Development and Validation of a Risk Score to Predict Unplanned Hospital Readmissions in ICU Survivors: A Data Linkage Study,” Australian Critical Care 18 (2023): 383–390, 10.1016/j.aucc.2023.05.002. 37339922

