# Supplementary material for: Swedish Consensus Recommendation for Follow‐Up After ICU Stay
Source: Acta Anaesthesiol Scand. 2026 Oct 25;71(1):e70361. doi: 10.1111/aas.70361 (PMC13696153; doi:10.1111/aas.70361)
Supplement: Supplementary file 1 [file AAS-71-0-s001.docx]

# Supplementary file 2

Search string Pubmed

#7,"Search (intensive care unit OR critical care) AND (intensive care unit OR ICU) AND (follow-up) AND (cognitive OR cognitive dysfunction) AND Humans [Mesh] AND adult [MeSH]))

Filters: Publication date from 2007/01/01 to 2024/12/31, AND ((review[Filter]) AND (english[Filter]) AND (alladult[Filter])

#6,"Search (intensive care unit OR critical care) AND (intensive care unit OR ICU) AND (follow-up) AND (psychological OR psychological distress) AND Humans[Mesh]

Filters: Publication date from 2007/01/01 to 2024/12/31, AND ((review[Filter]) AND (english[Filter]) AND (alladult[Filter])

#5,"Search (intensive care unit OR critical care) AND (intensive care unit OR ICU) AND (follow-up) AND (physical OR physical ability)” AND Humans[Mesh]

Filters: Publication date from 2007/01/01 to 2024/12/31, AND ((review[Filter]) AND (english[Filter]) AND (alladult[Filter])

#4,"Search (intensive care OR critical care) AND (intensive care unit OR ICU) AND (follow-up)” Filters: Publication date from 2007/01/01 to 2024/12/31, AND ((review[Filter]) AND (english[Filter]) AND (alladult[Filter])

#3,"Search (intensive care OR critical care) AND (intensive care unit OR ICU)”

Filters: Publication date from 2007/01/01 to 2024/12/31, AND ((review[Filter]) AND (english[Filter]) AND (alladult[Filter])

#2,"Search (intensive care OR critical care)” Filters: Publication date from 2007/01/01 to 2024/12/31, AND ((review[Filter]) AND (english[Filter]) AND (alladult[Filter])

#1,"Search (intensive care OR critical care)”
