# Supplementary material for: Swedish Consensus Recommendation for Follow‐Up After ICU Stay
Source: Acta Anaesthesiol Scand. 2026 Oct 25;71(1):e70361. doi: 10.1111/aas.70361 (PMC13696153; doi:10.1111/aas.70361)
Supplement: Supplementary file 2 [file AAS-71-0-s002.docx]

| Suppl.4 CASP - Methodological quality appraisal of each included study. |  |  |  |  |  |  |  |  |  |  |  |  |
| --- | --- | --- | --- | --- | --- | --- | --- | --- | --- | --- | --- | --- |
| Authors | Item 1 | Item 2 | Item 3 | Item 4 | Item 5 | Item 6 | Item 7 | Item 8 | Item 9 | Item10 | Decision | Quality |
|  |  |  |  |  |  |  |  |  |  |  |  |  |
|  |  |  |  |  |  |  |  |  |  |  |  |  |
| Jonasdottir et al 2015 | YY /N | Y | YY/N | YY/N | Y | YY/N | Y/NN | Y | Y | Y | Included | High |
|  |  |  |  |  |  |  |  |  |  |  |  |  |
| Jensen et al 2015 | Y | Y | Y | Y | Y | Y | Y | Y | Y | Y | Included | High |
|  |  |  |  |  |  |  |  |  |  |  |  |  |
| Schofield-Robinson et al 2018 | Y | Y | Y | Y | Y | Y | Y | Y | Y | Y | Included | High |
|  |  |  |  |  |  |  |  |  |  |  |  |  |
| Josepha op´t Hoog et al2021 | Y | Y | YY/N | Y | YY/C | Y | Y | Y | YY/N | Y | Included | High |
|  |  |  |  |  |  |  |  |  |  |  |  |  |
|  |  |  |  |  |  |  |  |  |  |  |  |  |
| met -Y = yes; not met - N = no; Unclear - C = cannot tell |  |  |  |  |  |  |  |  |  |  |  |  |
