# Supplementary material for: Swedish Consensus Recommendation for Follow‐Up After ICU Stay
Source: Acta Anaesthesiol Scand. 2026 Oct 25;71(1):e70361. doi: 10.1111/aas.70361 (PMC13696153; doi:10.1111/aas.70361)
Supplement: Supplementary file 4 [file AAS-71-0-s004.docx]

Supplementary Material 3. Reviewed articles

| **Authors** | **Year** | **Purpose** | **Results** | **Recommendations** | **Descision** |
| --- | --- | --- | --- | --- | --- |
| Butcher B, Eaton T, Montgomery-Yates A, Sevin C | 2022 | Identify the 3 categories of disability commonly associated with PICS and potential screening tests.  Describe several ways in which ICU follow-up clinics can benefit patients, their families, and the health care system.  List some ways in which ICU in person follow-up clinics differ from primarily telemedicine-based ICU follow-up clinics. | Because of variable resources and goals of health care institutions, there is no standard recipe for sustaining an ICU follow-up clinic. | A framework that includes securing adequate space; assembling an invested, multidisciplinary staff; procuring financial, information technology, and physical stuff; using screening tools to identify patients most likely to benefit and to identify disabilities; and selling it to colleagues, hospital administrators, and the community can increase the likelihood of success. | Excluded No systematic review |
| Josepha op´t Hoog SAJ, Eskes AM, van Mersbergen-de Bruin MPJ, Pelgrim T, van der Hoeven H, Vermeulen H, Vloet LCM | 2021 | Assess the effectiveness of ICU initiated transitional care interventions for patients and families on elements of PICS and/or PICS-F | No significant differences in favor of transitional care interventions on physical or psychological aspects of PICS-(F). One study with a nurse-led structured follow-up program showed a significant difference in physical function at 3 months. | A clear adapted framework or model may be helpful to share more evidence-based intervention strategies to offer continuity of care to ICU patients and families. | Included |
| Profitt T, Menzies V | 2019 | Examine the literature for studies that explored relationships among two or more of the variables cognitive, physical, and psychological symptoms to gain a better understanding of PICS | No study explored associations among all three variables. Inconsistence was found in the study designs, follow-up time frames, patient populations, and measures used. | Using well-designed methodologies and standardized instruments in a variety of ICU-survivor populations has the potential to contribute to the development of screening guidelines, preventative strategies, and treatments for clinically meaningful condition. | Excluded No follow-up |
| Schofield-Robinson OJ, Lewis SR, Smith AF, McPeake J, Alderson P | 2018 | Assess the effectiveness of follow-up services for ICU survivors that aim to identify and address unmet health needs related to the ICU period (HRQoL, mortality, depression and anxiety, PTSD, physical function, cognitive function, ability to return to work or education). | There was insufficient evidence, from a limited number of studies, to determine whether ICU follow-up services are effective in identifying and addressing the unmet health needs of ICU survivors. | Future studies shall be designed with robust methods and consider only one variable (the follow-up service) compared to standard care; this would increase confidence that the effect is due to the follow-up service rather than concomitant therapies. | Included |
| Jensen JF, Thomsen T, Overgaard D, bestle MH, Christensen D, Egerod I | 2015 | To evaluate the impact of routine follow-up consultations versus standard of care for ICU survivors | Follow-up consultations might reduce symptoms of PTSD by 3-6 months after ICU discharge in ICU survivors, but without affecting HRQoL and other outcomes investigated. | Planning of future RCTs should aim to standardize interventions and outcome measures to allow for comparisons across studies. | Included |
| Jonasdottir R, Klinke M, Jonsdottir H | 2015 | To analyze the structure, content, type of outcome variables and advantages of nurse-led ICU follow-up | Ward visits, phone consultations within the first week of hospital discharge, outpatient clinic two-three months post ICU (to primarily assess physical, psychological and social health and secondarily to help patients reflect on their ICU experience and revisit the ICU) availability of ICU follow-up until six months after ICU discharge. | Patients’ referral to different professions is central. | Included  Integrated review |
| Jones C | 2014 | To evaluate the impact of routine follow-up consultations versus standard of care for ICU survivors | Existing evidence indicated that follow-up consultations do not improve HRQoL, anxiety, depression, physical and cognitive function, and return to work, but appear to reduce symptoms of PTSD after ICU admission. | Post-ICU follow-up needs to be developed in collaboration with patients and their families, and the effect should be investigated in larger studies and within comparable settings. | Excluded  No systematic review |
| Rattray J | 2013 | To illustrate the potential physical and psychological problems faced by patients after critical illness, highlight some of the interventions that have been tested | Findings from international studies of a range of potential interventions are presented. However, establishing effectiveness for most of these still has to be empirically demonstrated. | There is need for coordinated, multidisciplinary, designated recovery and rehabilitation pathway that begins as soon as the patient is admitted into an ICU. | Excluded  No systematic review |
| Wilcox E, Brummel N, Archer K, Ely W, Jackson J, Hopkins R | 2013 | To summarize the existing literature on the prevalence, mechanisms, risk factors, and prediction of cognitive impairment after surviving critical illness. | Depending on the exact clinical subgroup, up to 100% of critical care survivors may suffer some degree of long-term cognitive impairment at hospital discharge; in approximately 50%, decrements in cognitive function will persist years later. | Suggest screening instruments. SF-36, psychological problems. | Excluded  No systematic review |
| Desai S, Law T, Needham D | 2011 | To describe complications after ICU treatment | Pulmonary, neuromuscular, cognitive, physical, and psychological problems are common after ICU. | Increasing the understanding of risk factors for PICS can help to identify patients who are most at risk and develop interventions to reduce the complications. | Excluded  No systematic review |
| Kiekkas P, Theodorakopoulou G, Spyratos F, Baltopoulos GI | 2010 | To conduct an overview of psychological distress associated with delusional ICU memories | Recall of delusional memories was associated with psychological distress symptoms. | Follow-up of high-risk patients and professional assistance may minimize the incidence and duration of PTSD symptoms. | Excluded  No systematic review |
| Herridge M | 2009 | To highlight physical and functional problems after ICU discharge | Rehabilitation should begin at ICU admission; risk stratify patients at ICU discharge and tailor both inpatient and outpatient rehabilitation according to their needs. | Suggests rehabilitation strategies for ICU survivors including; screening for problems (ADL, 6MWT, Barthel Index, HRQL, HADS, BDI-II, IES), optimizing ADL and independence for earlier return to work. | Excluded  No systematic review |
| Rattray J, Hull A | 2008 | Describe interventions that might  improve this aspect of recovery from intensive care | Intensive care can result in important emotional problems for a number of patients and that these may have an impact on subsequent recovery and may result in increased  use of health service resources. | Standardization of measures  used to assess outcome from intensive care and their validation in this population would add greatly to our understanding. | Excluded  No systematic review |
